# Confusion over live/dead stainings for the detection of vital microorganisms in oral biofilms - which stain is suitable?

**DOI:** 10.1186/1472-6831-14-2

**Published:** 2014-01-11

**Authors:** Lutz Netuschil, Thorsten M Auschill, Anton Sculean, Nicole B Arweiler

**Affiliations:** 1 Department of Periodontology, Dental School, Philipps-University Marburg, Marburg, Germany; 2 praxisHochschule, Cologne, Germany; 3 Department of Periodontology, Dental School, University of Berne, Berne, Switzerland

**Keywords:** Dental plaque, Biofilm, Microorganisms, Viability state, Vital fluorescence, Bacterial viability kit, Mutagenicity

## Abstract

**Background:**

There is confusion over the definition of the term “viability state(s)” of microorganisms. “Viability staining” or “vital staining techniques” are used to distinguish live from dead bacteria. These stainings, first established on planctonic bacteria, may have serious shortcomings when applied to multispecies biofilms. Results of staining techniques should be compared with appropriate microbiological data.

**Discussion:**

Many terms describe “vitality states” of microorganisms, however, several of them are misleading. Authors define “viable” as “capable to grow”. Accordingly, staining methods are substitutes, since no staining can prove viability.

The reliability of a commercial “viability” staining assay (Molecular Probes) is discussed based on the corresponding product information sheet: (I) Staining principle; (II) Concentrations of bacteria*;* (III) Calculation of live/dead proportions *in vitro.* Results of the “viability” kit are dependent on the stains’ concentration and on their relation to the number of bacteria in the test. Generally this staining system is not suitable for multispecies biofilms, thus incorrect statements have been published by users of this technique.

To compare the results of the staining with bacterial parameters appropriate techniques should be selected. The assessment of Colony Forming Units is insufficient, rather the calculation of Plating Efficiency is necessary. Vital fluorescence staining with Fluorescein Diacetate and Ethidium Bromide seems to be the best proven and suitable method in biofilm research.

Regarding the mutagenicity of staining components users should be aware that not only Ethidium Bromide might be harmful, but also a variety of other substances of which the toxicity and mutagenicity is not reported.

**Summary:**

– The nomenclature regarding “viability” and “vitality” should be used carefully.

– The manual of the commercial “viability” kit itself points out that the kit is not suitable for natural multispecies biofilm research, as supported by an array of literature.

– Results obtained with various stains are influenced by the relationship between bacterial counts and the amount of stain used in the test. Corresponding vitality data are prone to artificial shifting.

– As microbiological parameter the Plating Efficiency should be used for comparison.

– Ethidium Bromide is mutagenic. Researchers should be aware that alternative staining compounds may also be or even are mutagenic.

## Background

The definition of the so-called “viability state(s)” of microorganisms has been a matter of confusion and discussion for decades (for a glimpse of the plethora of literature see [1-5]). Recently two manuscripts discussed this topic. Hannig et al. [6] investigated the influence of a novel mouthwash containing hydroxyapatite microclusters on bacterial adherence by using the BacLight™ live/dead staining technique. Tawakoli et al. [7] compared different live/dead stainings *“for detection and quantification of adherent microorganisms”* in the initial oral biofilm. In line with earlier literature these two articles demonstrate that serious attempts have been made in the past decades to define the different states between dead and live (marine and oral) microorganisms, and that “viability staining” or “vital staining techniques” have been and are still used as a trial to overcome the problem of distinguishing between live and dead microorganisms in biofilms.

In recent years more and more scientists in dental biofilm research have become familiar with commercially available vitality/viability stains, especially the BacLight Assay (BLA; BacLight™ live/dead staining technique). However, this staining principle has severe shortcomings when applied to undefined natural multispecies biofilm samples. Results of this and other staining techniques should be compared to classical microbiological techniques like the assessment of colony forming units (CFU) and, more reliable, the calculation of bacterial plating efficiency (PE). These comparisons with a “gold standard” are quite rare when commercial kits are used in biofilm research. Furthermore, components of these vital stains may be potentially mutagenic.

In summary, the purpose of this manuscript is to debate the basis, usefulness and the risk of “viable” and “vital” stains especially in biofilm research, with specific attention to natural dental biofilms and vital fluorescence staining with Fluorescein Diacetate/Ethidium Bromide [8]. From a scientific point of view, it is important that data derived from such staining techniques should reflect the bacterial status correctly.

## Discussion

### What is the root of the problem?

From a holistic point of view the debate covers different levels. First, the discrimination of dead or alive microorganisms represents a crucial problem in (environmental) bacteriology. This basic problem has existed for decades and has not yet been solved. In this respect, the terms “vitality” and “viability” are often used and quite often mixed - some researchers completely interchange these terms [9].

Second, “vital stains” are generally only surrogates, but are quick and simple devices in studies examining, for example, the antibacterial effect of substances. Here the problem is the large variety of staining substances and thus of staining principles, so that the *“plethora of choices adds to confusion”*[10]*.* Similarly Pamp et al. [11] state: *“More recently developed stains, such as the Syto stains …. can efficiently stain cells in virtually any color of the rainbow”.* That might sound humorous – but merely reflects the problem. As just mentioned, Tawakoli et al. [7] used combinations of several stains (for example FDA, cFDA, TCFDA, EB, as well as SYTO 9/PI, Sytox red, besides Calcein AM). Davey [10] also refers to FDA, PI and SYTO 9, but moreover to substances like SYTO green I, DIBAC4, pyronine Y, rhodamine 123 and thiazole orange. It is no wonder that this author [10] refers to another four reviews only to inform about the *“modus operandi”* of the different fluorescent stains and the *“huge diversity of possibilities in terms of stain selection, concentration, staining time, etc”.*

When analyzing further, the problem becomes even more complex. Some authors criticize the limited use of propidium iodide (PI) as a cell viability *(sic!)* indicator [12,13], while others monitored striking differences between SYTO 9 and SYTO 12 regarding the influence of porins on uptake kinetics of these dyes [14]. This means that when antibacterial substances disturbing the cell membrane integrity are assessed the use of those vital staining techniques may be misleading. An inherent aspect of this problem, namely the suitability of staining methods, is the dependency on the stains’ concentrations of the results (see later).

Third, the users are for the most part unfortunately unaware that such “seductive” tests have only been validated for a very limited number of bacterial species [13,15]. From 15 000 “hits” (250 reviews) generated in PubMed by asking for “flow cytometry & bacteria”, only three were left after filtration of the database when using “biofilm” as an additional tracing term. None of this three articles contributes to the debate.

It is crucial that “vital stains” and, much more important, their combinations are directly compared to conventional bacteriological data. As discussed later in detail this cannot be the assessment of CFU, but of PE. In dentistry some corresponding work has been completed using single or a lucid number of species *in vitro* without conducting bacteriological tests [6,16-18], while some companies used this staining method trusting *per se* in its reliability [19,20]. When the SYTO 9/PI combination was in fact related to corresponding microbiological assessments the outcome was inconsistent [21-23]. In our opinion it is impossible to find out studies where particular examples of these vital stains and its combinations were properly compared and related to microbiological data concerning natural multispecies biofilms like dental plaque.

Finally, the dyes may be or are toxic or mutagenic. With respect to the list of compounds, as previously mentioned and excerpted from [7] and [10], it must be determined whether there is a harm or risk in the use of these substances.

### Viability versus Vitality

Netuschil [8] recorded 49 terms to describe “vitality states” of microorganisms (for example: active microbes, cryptic growth, direct viable count [DVC], progressive dormancy, vegetative dormancy, dwarf cells, moribund cells, nonculturability, nonplateable, stasis survival, reproductive viability, viable but not culturable [VBNC], non-viable but resuscitable, vital, viviform, etc.) as cited in 34 different corresponding publications [1,2,4,24-54] (cf. Table 1). While the table displays references from 1962 up to 1998, the debate is older and was already relevant at the turn of the 19^th^ to the 20^th^ century [55-60]. One example is the “Great Plate Count Anomaly” [61,62] see also [63]. Even at that time vital stainings were debated to be used as a trial to overcome the shortcomings of culture techniques [64-68]. It seems that the past discussion [69] was “revitalized” at the turn of this century [41,70-78]. Of note is that some terms (e.g., dormant, VBNC) are even relevant when referring to probiotic bacteria [79,80].

**Table 1:** **Terms used to describe “vitality states” of microorganisms (from**[8]**)**

	
Acclimation [30]	“Quiescent cells” [17]
Active microbes [20]	Resuscitation [3,4,15,24,28-31]
Alive, “aliveness” [15]	“Shut down cells”, “shut down state” [17]
“Anabiotic (dormant) state” [15]	Somnicells [8,11,30]
“Bags of enzymes” [4,12]	Starvation [10,17,29]
Cryptic growth [24,26,27,29]	– “True starvation” [15]
Culturable, culturability [4,10,11,31]	“Substrate accelerated death”, “substrate
– Nonculturable, nonculturability [22,24]	Accelerated stress” [6,15,26,29,31,33]
Debilitation [9]	Survival [5,11,20,22,29]
Dead, death [3,4,9,11,15,16,20,26],[29]	– “Survivability” [3]
– “Death phase” [15,29]	– “Stasis survival” [25]
“Die-off” [31]	Viable [3,10,15,16,18,30,31]
Direct viable count (DVC), DVC method [3,4,10,15,18,29-31,34]	– Non viable [3,15,16,20]
	Viability [3,4,6,9,12,13,15,16],[19,22,23,26,29]
Dormant, dormancy [11,12,15,20,23,28-30,32]	– “Apparent viability” [6,15]
– “Progressive dormancy” [1,30]	– “Reproductive viability” [23]
– “Vegetative dormancy” [15]	– “True viability” [6]
Dwarf cells, inactive dwarfs, ultramicrobacteria [6,14,15,29,32]	“Viable but nonculturable” (VBNC) [3,4,8,10,12,15,16,21],[22]
Growth arrest [25]	– VBNC hypothesis [2-4,7]
“Killer phenotype” [15]	– VBNC state [3,4,10,22,24,29,31,33],[34]
Lysis [20]	– “Viable but nonrecoverable” [31]
Moribund cells [29]	– “Non-viable but resuscitable” [16]
'Nonplateable" [24]	– “Unculturable but viable” [28]
Protistan grazing [11]	Vital, vitality [15,16,26]
“Pseudosenescent” [29]	Viviform [11,30]
**References:**	
^1^Barcina et al. 1989 [24]	^18^Kogure et al. 1979 [40]
^2^Barer et al. 1993 [25]	^19^Korber et al. 1996 [41]
^3^Bogosian et al. 1996 [26]	^20^Mason et al. 1986 [1]
^4^Bogosian et al. 1998 [27]	^21^McKay 1992[42]
^5^Bowden & Hamilton 1998 [28]	^22^Morgan et al. 1993 [43]
^6^Button et al. 1993 [29]	^23^Nebe-von Caron et al. 1998 [44]
^7^Colwell 1993[30]	^24^Nilsson et al. 1991 [45]
^8^Colwell et al. 1985[31]	^25^Nyström 1995[46]
^9^Dawe & Penrose 1978[32]	^26^Postgate 1977[47]
^10^Duncan et al. 1994[33]	^27^Postgate & Hunter 1962[48]
^11^Gonzáles et al. 1992[34]	^28^Rose et al. 1990 [49]
^12^Gribbon & Barer 1995 [35]	^29^Roszak & Colwell 1987a [2]
^13^Höfle 1983[36]	^30^Roszak & Colwell 1987b [50]
^14^Hood et al. 1987 [37]	^31^Roszak et al. 1984 [51]
^15^Kaprelyants et al. 1993 [4]	^32^Stevenson 1978[52]
^16^Kell et al. 1991 [38]	^33^Whitesides & Oliver 1997[53]
^17^Koch 1996 [39]	^34^Wilson & Lindow 1992 [54]

Unfortunately, several of the terms found and used in publications are incorrect, misleading or even paradoxical, especially the often used term “viable but not culturable (VBNC)”, which blurs the line between vitality and viability. To minimize confusion as much as possible we refer to Kaprelyants et al. [4]: *“Several classifications of the physiological states of microorganisms have been presented. We have previously suggested*[3]*that all the cell types considered could be reduced to three groups, as follows: ‘viable’ to refer to a cell which can form a colony on an agar plate, ‘vital’ to refer to one which can only do so after resuscitation and ‘non-viable’ to refer to a cell which cannot do so under any tested condition. According to this terminology, dormant cells are vital”* (see Table 2).

**Table 2:** **“Glossary of terms used to describe the 3 major physiological states defined herein” (cited from**[3]**)**

***Physiological state***	***Phenotype***
*Viable*	*Capable of division; will form a colony on an agar plate.*
*Vital or dormant*	*Unable to divide or to form a colony on an agar plate without a preceding resuscitation phase.*
*Non-viable*	*Incapable of division; will not form a colony on an agar plate under any tested condition.*

We use the phrases ‘starvation’ or ‘starving cells’ to refer to the environmental conditions under which cells are incubated, rather than to a physiological state. Thus starved cells (or cells that have suffered other stresses) may or may not be dormant. Despite historical usage of these terms, the phrases ‘direct viable count’ and ‘viable-but-non-culturable’ are misnomers, since such cells are not viable as defined above.

In accordance with [4] we define “viable” strictly as “capable to grow”. In this respect any other tests, for example elongation tests (DVC; [50]) or staining methods, are merely proxies, since no kind of staining can prove viability. Thus, the term “viability stain” is a misnomer *per definitionem* and these stains should correctly be named “vital stains”. Unfortunately, the misnomer is frequently used by Invitrogen Ltd. (BacLight™), and is consequently – but incorrectly – adopted by the users of these vitality tests.

### The BacLight™ bacterial viability kit (BacLight Assay, BLA)

According to the manufacturer [81] BLA consists of two stains, propidium iodide (PI) and SYTO 9, which both stain nucleic acids. SYTO9 is a green fluorescing intercalating membrane permeable molecule and stains all cells. In contrast, PI is a red intercalating stain and is membrane impermeable, and is therefore excluded by “healthy” cells. The manufacturer describes that PI has a stronger affinity to nucleic acids than SYTO 9; thus, when both stains are present within a cell, SYTO 9 will be displaced from nucleic acids and the cell(s) will fluoresce in red. To prove the mechanism Stocks [82] conducted “cell-free” physicochemical measurements with the “Viability Stain, *Bac*Light”, and could reveal that the staining principle is not that simple. This author established a so-called fluorescence resonance energy transfer (FRET), though, under certain staining conditions, SYTO 9 emission surpasses the PI emission. Increasing the SYTO 9 concentration may thus enhance the PI emission, causing a double staining of cells. Stocks [82] emphasizes several times that for an interpretation of the staining outcome *“the relative concentrations of PI, SYTO9 and DNA were of crucial importance”* and *“that appropriate control or validation experiments (should be) performed”.* Double staining and/or FRET was also documented by other authors [41,83], who examined viability parameters of viable and formaldehyde-killed or UVA-treated cultures, respectively. The following discussion of the MOLECULAR PROBES manual should be viewed in this context.

Concerning the reliability of the “viability kit” used in biofilm research we would like to directly cite the product information sheet(s) of MOLECULAR PROBES 2001; Product Information LIVE/DEAD ® BacLight™ Bacterial Viability Kit, Revised: 26-January-2001, as well as Revised: 15-July-2004 (whereby the latter represents the most current version in October 2013) [81]:

(I) *“… stains differ both in their spectral characteristics and their ability to penetrate healthy bacterial cells. When used alone, the SYTO 9 stain generally labels all bacteria in a population – those with intact membranes and those with damaged membranes. In contrast propidium iodide penetrates only bacteria with damaged membranes, causing a reduction in the SYTO 9 stain when both dyes are present. Thus, with an appropriate mixture of the SYTO 9 and propidium iodide stains, bacteria with intact cell membranes stain fluorescent green, whereas bacteria with damaged membranes stain fluorescent red."*

(II) *“Staining Bacteria with either Kit L7007 or L7012 - 4.1 Adjust the E. coli suspensions (live and killed) to 1 × 10*^*8*^*bacteria/mL or the S. aureus suspensions (live and killed) to 1 × 10*^*7*^*bacteria/mL. S. aureus suspensions typically should be 10-fold less concentrated than E. coli for fluorescence microscopy.”* Hence the numbers of *E. coli* and *S. aureus* differ by one logarithm.

(III) Lastly, due to **(I)** a mixture of 50% living and 50% dead bacteria does not normally lead to a 50/50 green/red fluorescence. And *vice versa:* 50% of green fluorescing bacteria in a sample does not mean that there are 50% vital cells. Therefore, *“green/red fluorescence ratios* (have to be) *calculated for each proportion of live/dead E. coli.”*

This last point was confirmed by Hannig et al. [6] in their paper concerning the viability of *S. mutans in vitro*. They determined that an *“initial concentration of viable bacteria in the assay”* of 50% leads to different *“ratio emission vital/emission dead bacteria”* values of about 9.5, 6.5 or 8.0 in their NaCl-control samples. Furthermore, Hannig et al. [6] state that *“the proportion of avital bacteria increased as indicated by the ratio of avital to vital cells. It ranged between 0.1… and … 29.0 … After rinsing with chlorhexidine, the ratio amounted to 190 (6 h) or 10.2 (12 h)…”* Taking the information of their Figure one into account, it remains unclear what these different ratios actually may mean in terms of “real” vitality values.

In summary, the three above-mentioned points described in the BLA user’s manual show that, in accordance with Stocks [82], an “*appropriate mixture*” of the two stains must be determined and established for any single species of bacteria before using them in an experiment. This may be possible in an *in vitro* situation, where the BLA may help to save time in the long run following critical, careful, and time consuming calibration steps for each individual bacterial species. However, this is impossible to manage in naturally occurring biofilms due to the uniqueness of the plaque matrix biofilms where in reality there may be more or less than 1000 different microbial species [84]. Moreover the application of the SYTO 9/PI stain is not considered suitable for biofilms, because of diffusion phenomena due to exo-polymers that result *“in an underestimation of viable counts”*[21].

However, there seems to be an additional drawback. Giertsen et al. [23] criticized considerable discrepancies between expected biofilm vitality and the outcome of the SYTO 9/PI staining method. Moreover, these authors described an instable behavior and a change of the staining color from green to red, *i.e.* towards monitoring more “dead” bacteria. Just recently these findings were explained by Tawakoli et al. [7] postulating that stained cells lost their viability shortly after intercalation of the dyes, writing: *“This hypothesis was confirmed by the TEM analysis in the current study (Figure two). The images showed immense lysis and destruction of the adherent cells ….”*

Table 3 lists a plethora of studies [5-7,9,16-20,22,23,41,71,73],[82,83,85-98] that used the BLA. However, in almost all studies it had to cleared up (a) if plaque-like biofilms or, at least, saliva samples were evaluated, or if the investigations were made with single bacterial species and/or artificial (monospecies) biofilms; (b) if the staining regime of the biofilm samples was adjusted according to the BLA manual (validation); (c) which dilution factor was used between SYTO 9 and PI, due to a validation procedure according to (b); (d) how the incubation procedure was followed, especially the incubation time of the bacterial samples together with the stains’ mixture; (e) whether the results of the BLA were compared to other parameters, and if the latter were appropriate (g) or not (f); (h) if the BLA results fit with the other parameters (whether appropriate or not). Last not least we were interested (i) whether the BLA results met the expectations of the users.

**Table 3:** Background information concerning the use of the BacLight® assay (BLA) for assessment of (dental) biofilm vitality

	
Studies using the BLA (n = 30)	[5-7,9,16-20,22,23,41,71,73],[82]* [83,85-98]
(a) Were plaque-like biofilms evaluated?	No: [5,16-18,22,23,41,82,83,86],[87,93,96-98]
	Yes: [6,7,9,19,20,71,73,85],[88-92,94,95]
(b) Validation	No: [5,7,9,16-18,20,22,23,41],[71,73,82,85-91,94-98]
	Yes: [6, 19?, 83, 92, 93?]
(c) Dilution factor	Not stated: [16,18,23,73,86,94,95,98]
	1:1 [6,7,9,17,20,22,41,71],[85,87-92,96,97]
	2:1 [93]
	4:1 [5]
	6:4 [19]
	1:6 [83]
(d) Incubation procedure	Not stated: [16,18,71,86,95,96]
	10 min, RT [6,7,20]
	15 min, RT [5,9,17,19,22,23,41,73],[85,87-91,97,98]
	20 min, RT [83,92]
	>20 min, 2°C [93]
	30 min [94]
(e) Comparison	No: [16-18,20,22,71,82,83,85,88],[90,91,94,95]
	Yes: [5-7,9,19,23,41,73,86,87],[89,92,93,96-98]
(f) … with inappropriate methods	[6,7,9,19,23,41,73,86],[87,89,92,93,96-98]
(g) … with appropriate methods	[5]*
(h) Did the BLA results fit to the other parameters?	No: [7,19,86,89,92,93] (DAPI), [96,97]
	Yes: [5,23,41,93] (FDA), [98]
(i) Did the BLA results meet the expectations of the user(s)?	No: [23,83,86]
	Yes: [5,7,16,18-20,22,41,71,82],[85,87-98]

(a) to (i) see description in the text.

Not stated: Either no information was given by the authors, or the authors stated that the staining was conducted “according to the manufacturer’s instructions”, what means generally a dilution factor of 1:1, and an incubation time of 15 minutes.

*[5]: Decker registered the total bacterial counts (as log BC/ml) and the CFU (as log CFU/ml), however gave no data regarding the PE.

*[82]: “cell-free” physicochemical measurements to elucidate the mechanism of the BacLight staining procedure.

From the 30 investigations listed in Table 3 one half (15) was classified in the rubric “plaque-like biofilm”. This high portion is due to the fact that we endeavored to consider literature concerning oral biofilms. Natural saliva was also included *e.g.*[89-91] as well as “microcosm plaques”, which were grown in an artificial mouth and/or were for example established from saliva [20,71,92] or from a subgingival plaque sample [96]. Some other studies dealt with deep-sea sediment bacteria or wastewater samples [73,93], which were considered by us as natural multispecies systems.

It is astonishing, but expected, that only five studies were based on a preceding validation procedure (line (b) in Table 3) [6,19,83,92,93], from which two cases were even questionable [19,93]. Nevertheless, a calibration could be assumed there. The description of Filoche et al. [92] clarifies the laborious methodology (cf. their Materials & Methods section, paragraphs 2.5 Generation of the viability standard; 2.6 Preparation of the individual plaque viability standard, 2.7 Preparation of the pooled viability standard; 2.8 Staining protocol for Live/Dead® BacLight™ and 2.9 Fluorescence measurement and data analysis). Surprisingly, the calibration procedure of these authors [92] even seemed to work when samples and controls were fixed in 4% paraformaldehyde, and/or were stored for up to three months.

As a consequence of having no validation the utmost users rely in the manufacturer’s advices regarding the dilution factor between the two stains SYTO 9 and PI. Only four research groups did not follow the recommended 1:1 dilution. Interestingly, their factors span a range from 1:6 up to 4:1 (line (c) in Table 3). This generally mirrors the seductive nature of the staining procedure [13] and the requests the researchers have towards an easy and quick application. The concern of Stocks [82] that the relative concentrations of PI, SYTO9 and the nucleic acids are of crucial importance is mostly neglected by the users.

At first glance the same holds true for the incubation procedure (line (d) in Table 3), however, this might be a more severe problem. Twenty-two of the users did not state the procedure or relied on the manufacturer’s instructions. Some others reduced the recommended 15-minute incubation to 10 minutes [6,7,20], while others extended the incubation time between the BacLight stains and their samples to 20 or even 30 minutes [83,92-94]. However, the finding of Tawakoli et al. [7] that the stained cells under investigation changed or even lost their viability shortly after intercalation of the dyes suggests that the “simple” incubation time is a crucial and potentially destructive factor. It is to question whether a time of 10, 20 or 30 minutes (“in the dark”, but at room temperature, and only once at 2°C [93]) may exert a deteriorating effect on the outcome of the staining procedure. This is of specific importance when the influence of antibacterial substances is assessed like the widely used and often studied chlorhexidine (CHX) or essential oils (EO), which affect the integrity of the bacterial cell membrane.

All three phenomena - FRET and double staining [41,82,83], potential impact of exopolymers [21], and the observation of decreasing vitality during the staining procedure [7] - point towards an overestimation of PI, *i.e.,* of dead cells. For an example Tomás and colleagues assessed the effects of CHX in saliva as measured with the aid of the BLA [89-91]. Especially 30 seconds after rinsing with CHX they revealed a very strong bactericidal action. However, their magnitude was in line with former (independent) conventional plating assays of the same research group [99]. Noteworthy, this research group had acceptable outcomes with the use of the staining procedures and presented convincing data regarding the antibacterial effect of different CHX concentrations, rinsing regimes [89,90] and influencing factors [91]. In sum, however, it cannot be cleared whether there is an artificial shifting towards “dead” values as long as no concomitant comparison with appropriate conventional parameters is made.

Different trials concerning comparisons were conducted by the BLA users (line (e) in Table 3), with the exception of [5] altogether with inappropriate methods. In our opinion (see next paragraph) only the plating efficiency (PE) as a relative parameter is appropriate, but not the CFU. Decker [5] registered the total bacterial counts (BC) as well as the CFU, both parameters being the mathematical basis to calculate the PE [8]. Nevertheless, she did not determine the corresponding PE values. Therefore, the positive and negative conclusions regarding the reliability of her different staining procedures cannot be justified.

Assessments of CFU were conducted by different authors, either in independent earlier publications [99-101] before the authors switched to the usage of the BLA [19,90,91], or simultaneously with their BLA measurements [7,19,23,86,89,92,96-98]. Quite astonishingly all these very different author groups tried to compare the relative parameter “percentage of vital bacteria”, as monitored by the BLA, with the absolute parameter CFU as assessed by plate counting. No wonder that the counts did not fit with the BLA in 7 of the 9 cases (line (h) in Table 3).

Finally, we tried to judge whether the authors were satisfied with the outcome of the BLA (line (i) in Table 3). This was more or less true in the majority of cases, independent of validation and comparison, and independent of the agreement of the BLA with the other (inappropriate) methods. Some authors were nearly delighted [92,93].

Taking all objective observations into consideration, incorrect statements were published by the users of the BLA. Some of the users [6,7,21,23,41,71,82,83],[86] even describe and discuss the shortcomings of their commercial stains. Similarly, as already mentioned, Davey [10] in her recent review criticizes some limitations or “stumbling blocks” in flow cytometry. No single stain or staining method has been found to be suitable for all organisms [102]. Consequently, the *modus operandi* of different fluorescent stains has even been described in several reviews (see for example [102-104]). The second limiting factor deserving consideration is the need for further method development and protocol adjustment, even when similar protocols have already been published. For example, microorganisms may need pretreatment, which may also be different for gram-positives and gram-negatives, such as the use of EDTA. Thus, such protocol modifications are necessary for each new bacterial species tested (“*strain- and matrix-specific optimization of the protocol*”) [10,97,105,106]. Again, it should be noted that the dental biofilm comprises, in a conventional view, of as many as or even more than 1000 diverse species [8,84] embedded in a complex matrix [8]. A current genetic analysis even discloses 10 000 species-level phylotypes [107].

### Colony Forming Units (CFU) and Plating Efficiency (PE) for comparison with vital stains

The only parameter that can be used for comparing the reliability of vital stains (of any kind) is the plating efficiency (PE). This is irrespective of the difficulties of determining “viability” via cultivation as mentioned above. PE can be calculated by relating plate counts (CFU) and total microscopic counts (MC), as conducted by Netuschil and coworkers with supragingival plaque biofilm bacteria *ex vivo*[108-110]. Table 4 (data taken from [8]) signifies a good relationship between Fluorescein Diacetate/Ethidium Bromide (FDA/EB) based vital fluorescence data (VF) and corresponding PE values. When PE and VF were assessed from the same plaque sample the PE resulted in lower values than VF (in 7 of 9 comparisons including about 500 samples, Table 4). This is to be expected because more microorganisms should be vital than viable.

**Table 4:** **Vital fluorescence (VF%) results compared to the corresponding bacteriological parameter plating efficiency (PE%) (data taken from**[8]**)**

**Reference**	**n**^**1**^	**Plaque age (Days)**	**VF (%) ± SD**	**Relation**^**2**^	**PE (%) ± SD**
Weiger et al. 1992 [108]	200	1	69.8 ± 16.0	n.a.^3^	60.4 ± 30.3
		2	78.0 ± 14.7	n.a.	91.9 ± 30.1
		3	81.3 ± 10.9	n.a.	82.4 ± 26.8
Weiger et al. 1994 [115]	132	1	42.9 ± 20.7	<	47.8 ± 21.8
		2	76.3 ± 17.5	>	58.8 ± 18.0
		3	86.3 ± 7.8	>	73.5 ± 30.6
von Ohle 1995 cf. [[109]]	211	1	57 ± 18	<	75 ± 35
		2	73 ± 20	>	53 ± 20
		3	79 ± 18	>	55 ± 20
Netuschil et al. 1995 [110]	160	1	52.1 ± 17.2	>	43.9 ± 27.2
		2	83.3 ± 12.9	>	77.3 ± 26.1
		3	90.8 ± 6.1	>	83.7 ± 17.0

^1^Number of independent plaque samples.

^2^Rough relation between VF(%) and PE(%).

^3^n.a.: not available – in this first study different plaque samples (however, from one patient each) were taken for assessment of either VF(%) or PE(%).

Moreover, Table 4 illustrates that initial dental plaque consists mainly of bacteria that are either not culturable or are dead. This has been proven by cultivation [111] or by cultivation and concomitant vital fluorescence (VF) [108-110]. In two hour old plaque samples Weiger et al. [109] calculated a mean PE of 30% compared to a mean value of 22% vital bacteria in VF (FDA/EB). In their study with 120 minute old adhering biofilm bacteria Tawakoli et al. [7] found vitalities ranging from 42% to 66% (cf. their Table 4).

PE data were not assessed in the investigation by Tawakoli et al. [7]. They discussed *“a certain variance between the different combinations”* of stains, which *“resulted in three groups: equal distribution [2 stains], more dead bacteria vs. viable [1 stain], more viable vs. dead bacteria [2 stains]”* (see the results in their article, Table 3)*.* However, without an accompanying calculation of PE an important question remains to be answered: Which of these (groups of) vital stains reflect (or do not reflect) the microbial reality? Similar to other authors [19,23,86,89,92,96-98], Tawakoli et al. [7] assessed the CFU, which only reflect the pure number of bacteria in their samples. No wonder that the latter authors state the following in their results: *“A correlation between the number of bacteria detected with CFU and the number of viable bacteria, detected with staining techniques, could not be observed.”*

### Dependence of the results of vitality testing on the stain’s concentration

Stocks [82] clearly stated that not only the relative concentrations of PI and SYTO9, but also their relationship to DNA are of crucial importance. This equals Acridine Orange (AO), which is falsely named a vital stain, and which stains nucleic acids either green (this was wrongly believed to be vital) or red (this was wrongly believed to be dead). It could easily be shown that dilutions or concentrations of only factor 2 lead to remarkable shifts in the green/red images [8]. This clarifies that the so-called “vitality values,” as assessed with AO, are dependent on its concentration, on pH and other factors, as well as on the relationship of the dye (whether in an adequate concentration or not) to the actual amount of stainable nucleic acids, DNA and/or RNA. Citations from the MOLECULAR PROBES Product Information [81], including **(I)** that an “*appropriate mixture*” of the dyes is necessary for reliable testing, and **(II)** that the number of bacteria to be tested has to be known and standardized in a species-specific manner, clarifies that the aforementioned statement concerning AO, in accordance with Stocks [82], also applies to the SYTO 9/PI stain.

However, no relevant concentration-dependency was found for the FDA/EB staining. Staining solutions containing the same basic concentration of FDA/EB were applied in different studies, where, due to differing study designs, the volumes of the used staining solutions ranged from 5 μl [112,113] to 50 μl [114], and even up to 500 μl [110,115], without changing the outcomes of the VF assessments [8].

The majority of the commercial staining components act via *passive* physicochemical distribution patterns, which are assumed to be different in (real) viable and (real) dead microbial cells. This also holds true for EB; however the color of EB cannot change due to concentration, pH or other physicochemical parameters [8]. In contrast, the non-fluorescent FDA penetrates the cell membranes of living cells, and is cleaved only in a *metabolically active* cell by different enzymes, mainly esterases [8,116,117], to yield the fluorescing Fluorescein. Thus, a functioning metabolism is a necessary prerequisite for positive intracellular (vital) staining. Similar to the red EB counter stain, the green Fluorescein staining is neither hampered nor changed by physicochemical effects.

### Vital fluorescence assessments in dental biofilm research

It is to question why the “traditional” VF stains of FDA and EB used in oral biofilm research should be replaced with other substances that exert a similar health risk (see next paragraph) and are not proven to be suitable and reliable in biofilm studies. Regarding FDA/EB, an assortment of existing publications (apart from numerous cell culture and cytotoxicity investigations) can be cited from research groups Netuschil [108-110,112,114,115,118-124], Brecx [125-128], Arweiler/Auschill [113,129-143] and others [144-155]. In this context the FDA/EB vital fluorescence staining was routinely used together with Confocal Laser Scanning Microscopy (CLSM) [119,124,131,134-136,139,140,143,153] to establish the three-dimensional vitality pattern of the (early) dental biofilm or to document the antibacterial effects of dental materials, mouthrinse solutions as well as food preservatives.

Worth noting is that the FDA/EB VF staining method discriminates very well between the bactericidal effects of diverse mouth rinse preparations [8,110,113,120,123,125,126,129],[133,137,138,145]. Also, due to easy handling and to the independency of calibration procedures as well as concentration and other physical and chemical parameters, the results obtained via FDA/EB staining can be compared between different studies and even between different research groups.

### Mutagenicity of staining solutions

Tawakoli et al. [7] argue that EB, which stains by intercalation in DNA, is mutagenic. Without any question, this fact has to be taken into consideration [8]. Because high amounts of EB are used in genetic research around the world, research laboratories are aware of this compound and its carcinogenicity. The authors of this article often experienced deep negativity towards EB, and only upon mentioning the name of this compound amongst the laboratory staff caused great concern. Nevertheless, at four different universities in Germany (Tübingen, Homburg/Saarland, Dresden and Freiburg, 1980 till 2009) we received general permission from the safety authorities to dispose our FDA/EB staining solutions in the normal waste due to the very scarce amounts of EB used.

The handling procedures of EB correspond with sources found in the internet [Wikipedia, Ethidium bromide, June 2012, see {further citations} there]: *“Ethidium bromide is not regulated as hazardous waste at low concentrations {17}.”* Due to its use in veterinary medicine as an anti-trypanosoma medicament {1}, its non-mutagenic effect in mice during a “*subchronic carcinogenicity study”* {11} and its effect even as an antitumorigenic chemotherapeutic agent {12} the *“above studies do not support the commonly held idea that ethidium bromide is a potent mutagen in humans….”* Nevertheless, Wikipedia recommends to be cautious and the “*material should be handled according to the material safety data sheet….”*

Ironically, the alternatives PI and the SYTO 9 are also potentially hazardous chemicals. Here also we would like to cite [81] the MOLECULAR PROBES Product Information **(IV)**: *“Storage and Handling: Caution: Propidium iodide and SYTO 9 stain bind to nucleic acids. Propidium iodide is a potential mutagen, and we have no data addressing the mutagenicity or toxicity of the SYTO 9 stain. Both reagents should be used with appropriate care …. As with all nucleic acid stains, solutions containing these reagents should be poured through activated charcoal before disposal. The charcoal must then be incinerated to destroy the dyes.”*

Thus, it seems that the difference between EB and other intercalating dyes (for an example see [156]) is not their potential mutagenicity, but the fact that the mutagenicity of EB has been commonly known for decades. In contrast, laboratory staff and other users of these chemicals are not aware of the similar risk that PI, SYTO 9 and other nucleic stains could have.

## Summary

Table 5 presents some “puzzle pieces” that are part of this debate. As pointed out (cf. Davey [10] as well as Pamp et al. [11]) a plethora of possibilities exist concerning vital stains, staining methods, and staining principles. For example, Tawakoli et al. [7] used combinations of several stains, which were in part related to the FDA/EB combination (FDA, cFDA, TCFDA and EB) or resembled more the BacLight drawer (SYTO 9/PI, Sytox red).

**Table 5:** Comparison of some staining principles in regard to their suitability for biofilm research

**Staining principle**	**Stain(s) (combinations)**	**Comparison with microbiological data (PE)**		**Concentration independency**	**Suitability for biofilm research**	**Potentially mutagenic**
		***Single species in vitro***	***Biofilm ex vivo or in situ***			
Vital fluorescence (FDA/EB) cf. [8,110,112]	Fluorescein diacetate, Ethidium bromide	+	++	Proven [8,110,112-115]	Proven (cf. Table 4)	+^1^
BacLight® (cf. Table 3)	Syto 9, Propidium iodide	**-**	**-**	Questionable (cf. Table 3)	Questionable (cf. Table 3)	+^2^
Staining according to [10,102]	Diverse substances	**?**	**?**	Non-existing or questionable	Non-existing or questionable	**?**

^1^Proven.

^2^Generally to assume, partly proven.

**?**Partly known or not known or not available from the diverse substances.

An inherent aspect concerning the suitability of staining methods is the dependency on the stains’ concentrations of the results. Table 4 lists the literature showing the independency of concentration of the FDA/EB vital staining. In contrast, the evidence does not seem to exist for a vast majority of the other stains.

It is compelling that “vital stains” (or however they might be named) and, even more important, their numerous combinations are directly comparable to appropriate conventional bacteriological data. This cannot be the assessment of CFU, but of PE. As depicted in Table 4 corresponding data exists for FDA/EB. To the best of our knowledge this does not hold true for the often used BLA (cf. Table 3 and corresponding text).

In summary, our concluding statements are as follows:

– The nomenclature regarding “viability” and “vitality” should be used with appropriate care. *Per definitionem* no kind of stain used for bacteria can prove their “viability”. Thus, such stains generally should be named “vital stains”.

– According to the BLA manual itself and the corresponding literature, the kit is not suitable for natural multispecies biofilms research. The kit is meant for use on a single defined bacterial species in a concentration of staining solution that was determined following a thorough calibration procedure.

– As a consequence of the kit's limitations, there is a strong assumption that the results obtained with several stains are influenced not only by physical and chemical parameters, but also by the relationship between total bacterial counts (viable, vital or dead) and the amount of the stain used in the test. Thus, the vitality data collected are prone to a completely unknown percentage of artificial shifting.

– Contrarily, no corresponding concentration-dependency (or “relationship-dependency”) was found with respect to FDA/EB. Moreover, the green intracellular (vital) Fluorescein staining originates only in metabolically active (bacterial) cells.

– Colony forming units (CFU) are not a useful parameter to compare to the results of “vitality staining”. Instead the plating efficiency (PE) should be used, if possible to conduct.

– It is a common belief that EB is highly mutagenic. However, the documented data is controversial. EB seems not to be a hazardous mutagen in humans. In this respect, researchers and laboratory staff should be aware that alternative staining compounds may also be or even are mutagenic.

## Competing interests

The authors declare that they have no competing interests.

## Authors’ contributions

NBA: Initiator of the review, vital fluorescence biofilm research regarding clinical controlled studies; LN: Main author, microbiology and `dead and alive´ discussion; TMA: CLSM research, Syto 9 and related compounds; AS: Mutagenicity of vital stains, refining and general support in editing. All authors read and approved the final manuscript.

## Pre-publication history

The pre-publication history for this paper can be accessed here:

http://www.biomedcentral.com/1472-6831/14/2/prepub
